# The Board Wins

**Published:** 1910-01

**Authors:** 


THE BOARD WINS.—Jewel P. Lightfoot, the friend of the medical profession, was inaugurated Attorney General of Texas January 1st to fill out Davidson's unexpired term. Among the very first things he did was to give an opinion sustaining the State Board of Health in its contention that under Section 10 of the act creating the Board they have the power to formulate and enforce a sanitary code in the interest of public health. That settles the vexed question whether or not the Legislature can delegate legislative powers to a board of health. Upon receipt of Judge Lightfoot's opinion the Governor signed the code and it will be promulgated and enforced forthwith. Lightfoot is broad-minded, able and zealous. Every doctor in Texas should vote for him next summer when he will be a candidate for Attorney General.

---

SEPARATE the membership fee in our State Association from the subscription fee to the *Journal,* and let subscription to the *Journal* be discretionary and not, as now, compulsory. Let the membership fee be $1 again and let those who wish the *Journal* say so, and subscribe for it. I shall introduce a resolution to that effect at Dallas. The American Medical Association has taken the initiative and done that very thing. See Advertising page 20, December 4th issue: "The annual assessment for membership shall be $1; and the price of subscription to the *Journal* shall be $4 for members."

---

UNINTENTIONAL humor is the most delicious. An editorial writer in the *Atlanta Journal Record of Medicine* says that if the government will change Atlanta from the area of Central time to that of Eastern time, it will "give Atlanta people one hour more of sunshine each day." That is, to set the clocks up an hour will make the sun rise an hour earlier at Atlanta. He doesn't say how it would affect those in the same longitude outside of Atlanta, nor where the sunshine would come from on rainy days. This plan is recommended (by us) to cities further west: San Francisco, for instance. By setting her clocks up, say, ten hours, the people could have almost perpetual sunshine. I heard, once, of a little bantam rooster that used to crow at a certain hour to let the sun know when to rise. He was sick one morning and didn't crow; and the sun overslept itself and didn't get up until way after breakfast.

